# Supplementary material for: Polyketide synthase gene clusters in the ericoid mycorrhizal fungus Oidiodendron maius: genome-wide prediction, expression profiling, and functional characterization of knockout mutants
Source: Front Plant Sci. 2026 Sep 16;17:1924430. doi: 10.3389/fpls.2026.1924430 (PMC13623903; doi:10.3389/fpls.2026.1924430)
Supplement: Supplementary file 1 [file SupplementaryFile1.zip › Supplementary Materials/Supplementary Folder 1.zip!/Oidma1_AssemblyScaffolds_Repeatmasked/knownclusterblast/cluster17/jgi.p_Oidma1_40859_mibig_hits.html]

| MIBiG Protein | Description | MIBiG Cluster | MiBiG Product | % ID | % Coverage | BLAST Score | E-value |
| --- | --- | --- | --- | --- | --- | --- | --- |
| ABB90284.1 | isoamyl\_alcohol\_oxidase | BGC0001057 | Zearalenone\_biosynthetic\_gene\_cluster | 29 | 101.02915952 | 240 | 3.7e-63 |
| AGA37266.1 | oxidoreductase | BGC0000816 | Malbrancheamide\_biosynthetic\_gene\_cluster | 29 | 99.3138936535 | 190 | 4.4e-48 |
| ADM34137.1 | oxidoreductase | BGC0001084 | Notoamide\_/\_stephacidin\_biosynthetic\_gene\_cluster | 28 | 99.1423670669 | 186 | 8.4e-47 |
| ANY57886.1 | PenH | BGC0001372 | Penigequinolone\_biosynthetic\_gene\_cluster | 28 | 97.4271012007 | 178 | 1.7e-44 |
| ABV57823.1 | oxidoreductase | BGC0001267 | Lysergic\_acid\_/\_elymoclavine\_biosynthetic\_gene\_cluster | 35 | 45.1114922813 | 135 | 1.7e-31 |
| KGO40470.1 | FAD\_linked\_oxidase,\_N-terminal | BGC0001205 | Communesin\_biosynthetic\_gene\_cluster | 32 | 38.5934819897 | 96 | 1.5e-19 |
